# Three novel methods to measure the postoperative displacement of lower urinary tract structures following radical prostatectomy in a sample of Korean patients

**DOI:** 10.1186/s12894-019-0472-6

**Published:** 2019-06-18

**Authors:** Hong Koo Ha, Henk B. Luiting, Petra L. Graham, Manish I. Patel, Jaspreet S. Sandhu, Oguz Akin, Sean F. Mungovan

**Affiliations:** 1 Department of Urology, Pusan National University Hospital, Busan, South Korea; 2 Pusan National University School of Medicine, Busan, South Korea; 3 Biomedical Research Institute, Pusan National University Hospital, Busan, South Korea; 4 The Clinical Research Institute, Sydney, Australia; 5 Faculty of Medical Sciences, University of Groningen, Groningen, The Netherlands; 6 Centre for Economic Impacts of Genomic Medicine (GenIMPACT), Macquarie University, Sydney, Australia; 7 Department of Urology, Westmead Hospital, Sydney, Australia; 8 Discipline of Surgery, Sydney Medical School, The University of Sydney, Sydney, Australia; 9 Urology Service, Department of Surgery, Memorial Sloan- Kettering Cancer Center, New York, USA; 10 Department of Radiology, Memorial Sloan- Kettering Cancer Center, New York, USA; 11 Westmead Private Physiotherapy Services, Westmead Private Hospital, Suite 6, 16-18 Mons Road, Westmead, NSW 2145 Australia; 12 Department of Health Professions, Faculty of Health, Arts and Design, Swinburne University of Technology, Melbourne, Australia

**Keywords:** Displacement, Measurement, Reliability, Urethra, Urinary tract, Magnetic resonance imaging

## Abstract

**Background:**

There is a change in the position of the remaining anatomical structures of the lower urinary tract system following radical prostatectomy. The aims of this investigation were to describe three novel methods used to measure the displacement of i) the vesico-urethral junction (VUJ), proximal membranous urethra (PMU) and anorectal junction (ARJ) and ii) the VUJ angle of displacement in men following radical prostatectomy and determine their intra- and interrater reliability.

**Methods:**

Retrospective comparative measurement of twenty pre- and postoperative MRI scans was undertaken by one observer on two separate occasions and on one occasion by another observer. Three standardized midsagittal pelvimetry reference lines were used to describe three X, Y axis measurement systems. The displacement (mm) of the VUJ, PMU and ARJ, and the angle of displacement (degrees) of the VUJ was measured for each of the three methods. Interrater reliability of VUJ, PMU and ARJ displacement and the VUJ angle of displacement measurements was assessed using a two-way mixed-effects agreement intra-class correlation coefficient (ICC) with 95% confidence intervals (CI). Test-retest (intrarater) reliability was calculated using a two-way random effects consistency ICC with 95% CI for all displacement measures of the VUJ, PMU and ARJ for one observer between two days.

**Results:**

The pubococcygeal line (PCL) axis measurement system demonstrated good to excellent intrarater and interrater reliability (ICC 95% interval lower bound > 0.75) for the VUJ and PMU displacement and the VUJ angle of displacement measurements. Other measurement systems were less reliable and more variable.

**Conclusions:**

In this sample of 20 Korean patients with median prostate volume 27.5 mL and maximum volume 70 mL, the measurement methodology using the PCL consistently demonstrated good to excellent reliability and the lowest variability for the measurement of the displacement of the VUJ and PMU and the VUJ angle of displacement. The PCL methodology is recommended as the method of choice. Further studies should validate these results in patients with large prostate volumes.

## Background

Radical prostatectomy (RP) is the primary surgical option for men with localised prostate cancer, providing definitive oncologic control. Following removal of the prostate gland, the position and function of the remaining anatomical structures of the lower urinary tract system are known to change. Typically, there is inferior displacement of the vesiculo-urethral junction (VUJ) and the intrapelvic displacement of the proximal membranous urethral stump (PMU), that allows for the formation of the urethrovesicular anastomosis [1, 2]. Despite modifications and improvements in the surgical technique, postoperative impairments of the lower urinary tract system, including urinary incontinence and erectile dysfunction occur in the majority of patients [3, 4].

Pre- and postoperative magnetic resonance imaging (MRI) can provide clinicians with high resolution and sharp depiction of the anatomical structures of the lower urinary tract system that are displaced following RP, including the VUJ, PMU and periprostatic structures such as the anorectal junction (ARJ) [5–8]. Preoperative MRI pelvimetry assessment has been used for surgical planning and to investigate the relationship between the position of the prostate and oncologic outcomes [9, 10]. Postoperative MRI pelvimetry assessment has reported lower urinary tract morphological changes within the pelvis in relation to continence outcomes [6, 7, 11, 12]. Similarly, using pre- and postoperative MRI images, changes in the size of anatomical structures including the membranous urethral length and variations in the thickness of the pelvic floor musculature following RP have also been reported [6, 7].

Quantifying the reliability of postoperative displacement (mm) of the VUJ, PMU and the ARJ and the direction of displacement (degrees) of the VUJ will allow the selection of the preferred measurement method for use in studies of the post-surgical displacement of anatomical structures and its relationship with urinary incontinence and erectile dysfunction. To our knowledge a reliability study of methods for measurement of anatomical displacement has not been reported.

In this investigation, we describe and assess the reliability of three new methods to measure the postoperative displacement of the VUJ, PMU and ARJ, and the postoperative angle of displacement of the VUJ.

## Methods

The aim of this investigation was to examine the test-retest and inter-rater reliability of three novel MRI measurements of postsurgical displacement of lower urinary tract structures.

### Patients

Following Pusan National University ethical approval (H-1707-009-057), we reviewed the pre-and three month postoperative MRI examinations of twenty randomly selected patients who underwent laparoscopic bladder neck sparing RP performed by a single surgeon (HKH). A simple random sample of patients was selected by using a computer to randomly shuffle identification numbers, then the first twenty numbers in the shuffled list were selected. Patient characteristics including age (years), preoperative prostate specific antigen (ng/mL), prostate volume (mL), membranous urethral length (MUL, mm) and Gleason grade grouping were recorded.

### Magnetic resonance imaging

Pre- and postoperative sagittal, axial and coronal T2W MRI images were captured using a 1.5 T or 3 T MRI unit (Magnetrom Symphony; Siemens, Erlangen, Germany) with the patient positioned in the supine position.

**Fig. 1:**
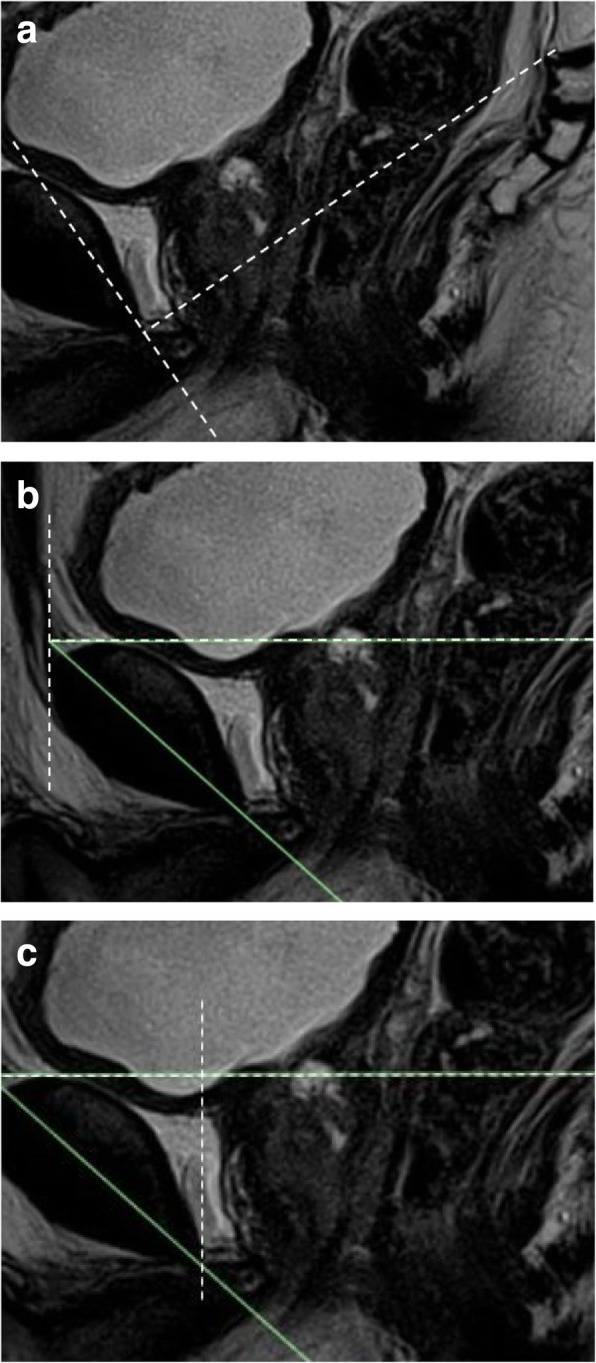
The three common axis systems that were investigated. **a** The pubococcygeal line (PCL) axis system was comprised of the line formed between the inferior rim of the pubic symphysis and the sacrococcygeal joint (PCL line) (X) and the perpendicular line to the PCL at the inferior rim of the pubic symphysis (Y). **b** The HOR-1 axis system was comprised of the horizontal line of the symphysis angle (X) and the line perpendicular to the horizontal line at the symphysis angle (Y). **c** The HOR-2 axis system was comprised of the horizontal line of the symphysis angle (X) and the line perpendicular to the horizontal line at the inferior rim of the pubic symphysis (Y)

We undertook the measurement of the pre- and postoperative MRI images for each patient using three standardised MRI measuring methods i) pubococcygeal line (PCL), ii) midsagittal horizontal line one (HOR-1) and iii) midsagittal horizontal line two (HOR- 2) (Fig. 1).

The measurement procedures were undertaken independently by two observers: on two separate occasions by one observer (HBL) and on a single occasion by another observer (SFM). Each of the three MRI measuring methods was used to conduct pelvimetry measurements of the postoperative displacement (mm) of the VUJ, PMU and the ARJ and the postoperative angle of displacement VUJ within the pelvic cavity.

### Pelvimetry measurements

#### Reference Lines

Pre- and postoperative PCL [13], HOR-1 and HOR- 2 [14] provided separate X, Y axis systems for each respective MRI measuring method (Fig. 1)

#### Anatomical Landmarks

**Fig. 2:**
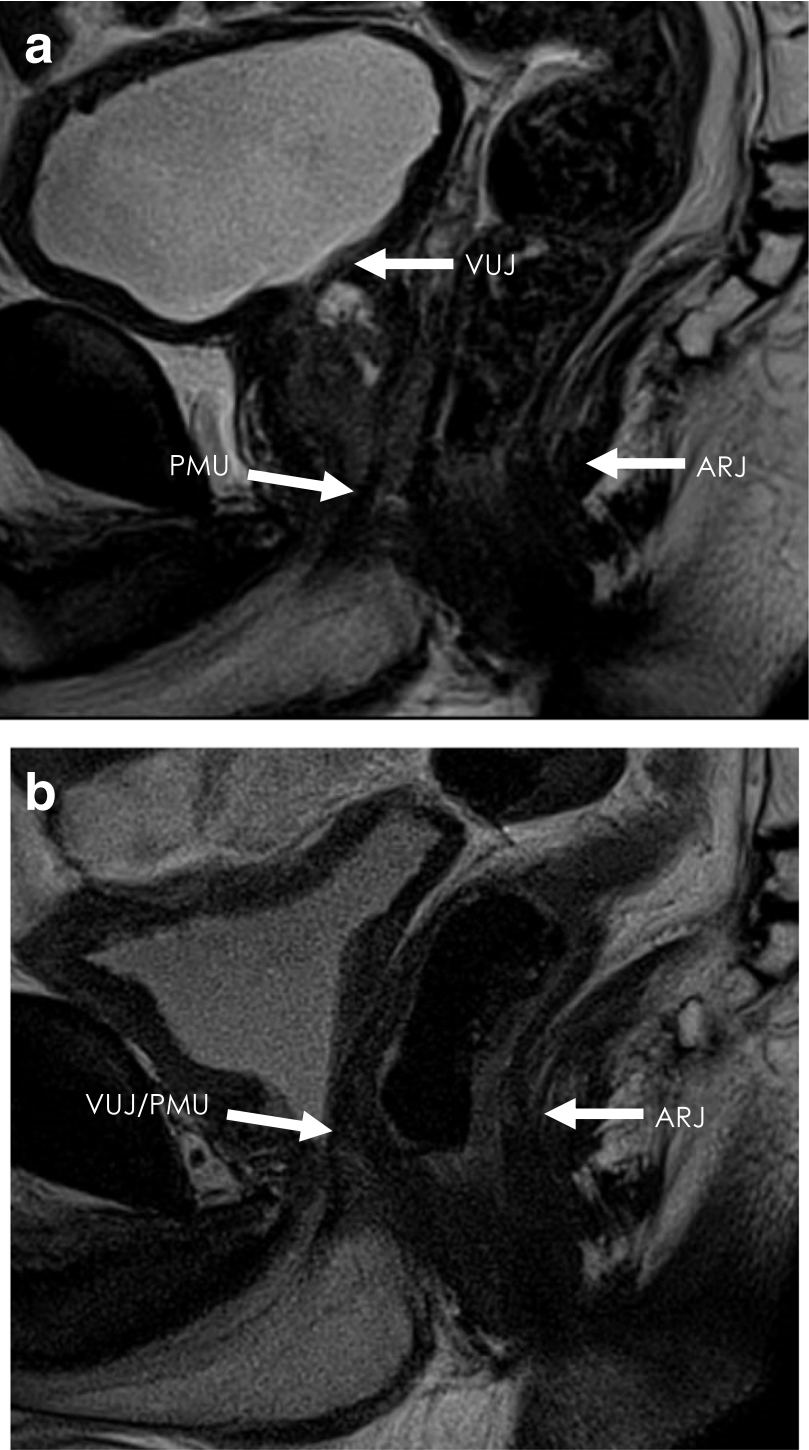
The **a**) preoperative and **b**) postoperative T2W midsagittal MRI images showing the VUJ, PMU and ARJ anatomical landmarks

The VUJ, PMU and ARJ were identified on the pre- and postoperative T2W midsagittal MRI images (Fig. 2). T2W coronal and axial images were used to confirm the position of the VUJ, PMU and ARJ on the midsagittal images.

#### VUJ

The preoperative VUJ was located at the bladder neck and was defined as the midpoint between the ventral and dorsal aspect of the VUJ (Fig. 2a). The postoperative VUJ was located at the most distal section of the bladder neck at the urethrovesicular anastomosis (Fig. 2b).

#### PMU

The preoperative PMU was located at the junction between the membranous urethra and the apex of the prostate (Fig. 2a), and the postoperative PMU was defined as the most proximal section of the membranous urethra at the VUJ (Fig. 2b).

#### ARJ

The ARJ was defined as the point of transition between the posterior wall of the rectum and the posterior wall of the anal canal. The ARJ was identified where there was an abrupt change in the orientation of the posterior wall of the rectum and the anal canal, where the puborectalis muscle could also be identified (Figs. 2a and b).

### Pelvimetry X, Y axis system measurements

**Fig. 3:**
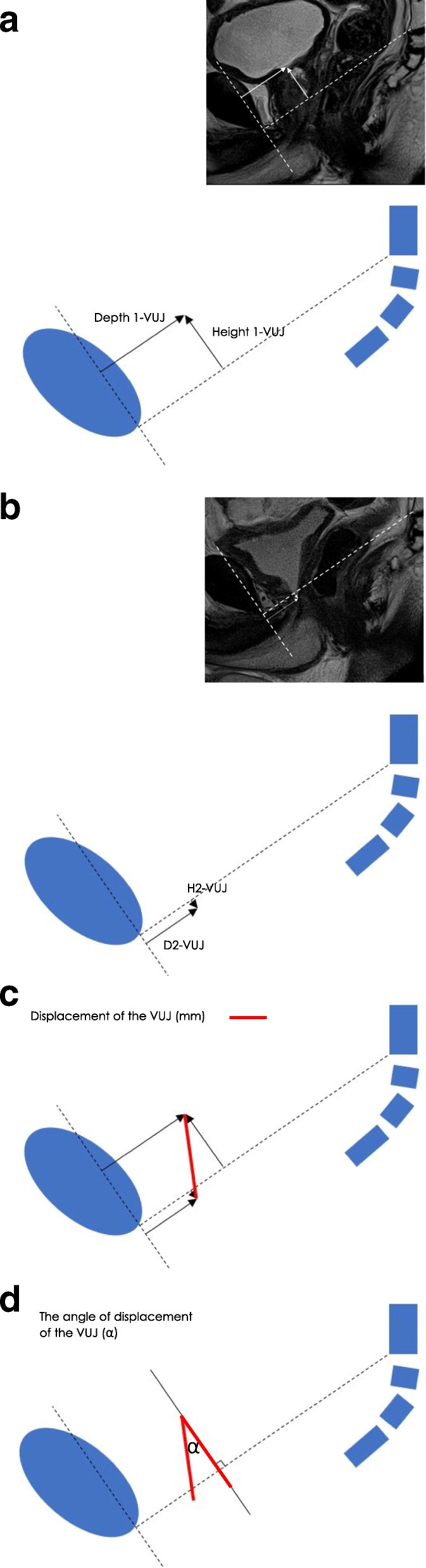
PCL Axis System: The **a**) preoperative VUJ (Depth 1-VUJ and Height 1-VUJ), **b**) postoperative VUJ (Depth 2-VUJ and Height 2-VUJ) **c**) VUJ postoperative displacement **d**) postoperative angle of displacement

**Fig. 4:**
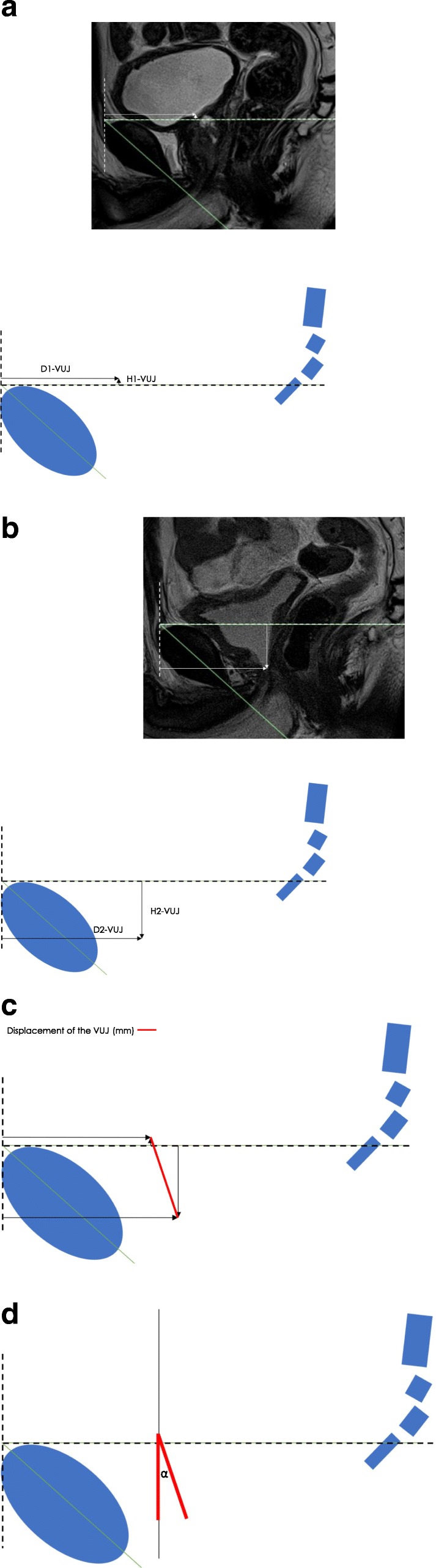
HOR-1 Axis System: The **a**) preoperative VUJ (Depth 1-VUJ and Height 1-VUJ), **b**) postoperative VUJ (Depth 2-VUJ and Height 2-VUJ) **c**) VUJ postoperative displacement **d**) postoperative angle of displacement

**Fig. 5:**
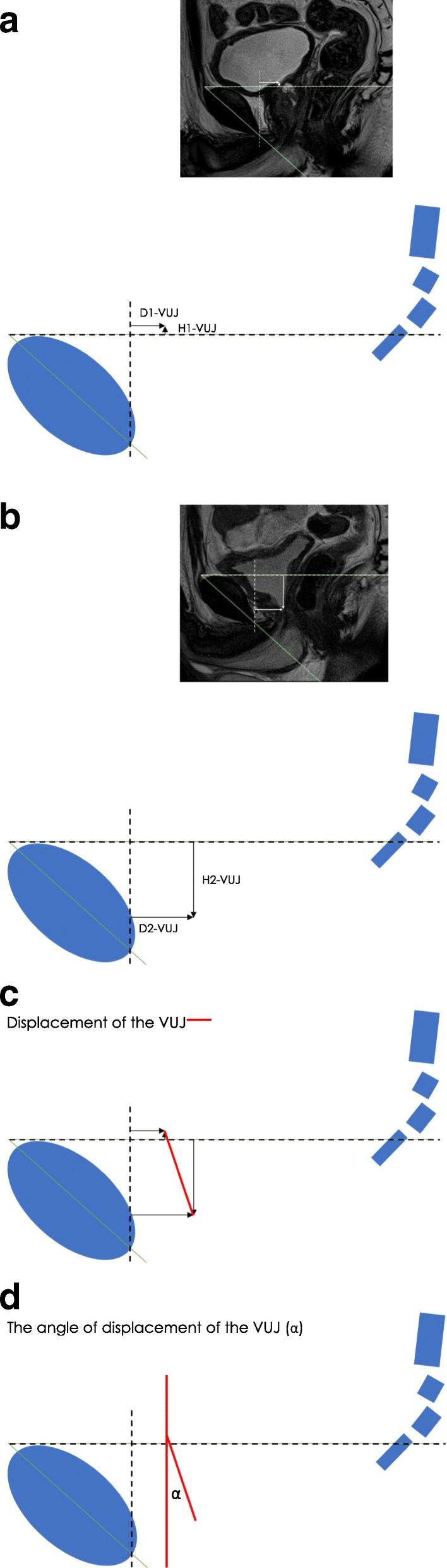
HOR-2 Axis System: The **a**) preoperative VUJ (Depth 1-VUJ and Height 1-VUJ), **b**) postoperative VUJ (Depth 2-VUJ and Height 2-VUJ) **c**) VUJ postoperative displacement **d**) postoperative angle of displacement

The pre- and postoperative horizontal and vertical positions of the VUJ, PMU and ARJ for the PCL (Fig. 3), HOR-1 (Fig. 4) and HOR-2 (Fig. 5) using the X, Y axis systems were determined.

#### X Axis

The horizontal measurement along each X axis reference line, i.e. the depth within the pelvis from the pubic symphysis was measured (mm). The pre- and postoperative depth of the VUJ (Depth 1-VUJ and Depth 2-VUJ), PMU (Depth 1-PMU and Depth 2-PMU) and the ARJ (Depth 1-ARJ and Depth 2-ARJ) were measured. The X axis measurements of Depth 1 and Depth 2 were positive integers for the PCL and HOR-1 axis systems (Figs. 3, 4). Depth 1 and Depth 2 measurements for the HOR-2 X axis were negative integers when anterior to the perpendicular Y axis and positive when posterior to the perpendicular Y axis (Fig. 5).

#### Y Axis

The vertical measurements along each Y axis reference line, i.e. the height within the pelvis perpendicular to the X axis reference line, were measured (mm). The pre- and the postoperative height of the VUJ (Height 1-VUJ and Height 2-VUJ), PMU (Height 1-PMU and Height 2-PMU) and the ARJ (Height 1-ARJ and Height 2-ARJ) were measured. Height 1 and Height 2 measurements above all three reference lines were recorded as positive integers and measurements below the three reference lines were recorded as negative integers (Figs. 3, 4, 5).

Standard DICOM viewer software (Intelerad Medical Systems, Canada) was used for all VUJ, PMU and ARJ depth (X axis) and height (Y axis) measurements. Training was given to members of the investigating team (SFM and HL) by an experienced radiologist (OA) and three urologists (HKH, JSS and MIP). The observers (SFM and HL) were blinded to all identifiable patient data when undertaking all measurements.

#### Postoperative displacement calculation

The following equation was developed to measure the postoperative displacement (mm) of the VUJ, PMU and ARJ for the PCL, HOR-1 and HOR-2 axis systems:

$$ \mathrm{Displacement}\ \left(\mathrm{mm}\right)=\sqrt{{\left(\  Depth\ 1- Depth\ 2\right)}^2+{\left( Height\ 1- Height\ 2\right)}^2} $$

Depth 1 = Preoperative depth (mm) of the VUJ (Depth 1-VUJ), PMU (Depth 1-PMU) and ARJ (Depth 1-ARJ); Depth 2 = Postoperative depth (mm) of the VUJ (Depth 2-VUJ), PMU (Depth 2-MU) and ARJ (Depth 2-ARJ); Height 1 = Preoperative height (mm) of the VUJ (Height 1-VUJ), PMU (Height 1-PMU) and ARJ (Height 1-ARJ); Height 2 = Postoperative height (mm) of the VUJ (Height 2-VUJ), PMU (Height 2-PMU) and ARJ (Height 2-ARJ).

#### Postoperative angle of displacement calculation

The postoperative angle of displacement (*α*) of the VUJ was calculated as the angle that was formed between the intersection of two lines: i) the line connecting preoperative and postoperative position of the VUJ and ii) the perpendicular line from the preoperative position of the VUJ to either the PCL, HOR-1 or HOR-2 (Figs. 3, 4, 5). The postoperative angle of displacement (*α*) of the VUJ was calculated for each of the three reference lines using the following equation.

$$ \mathrm{VUJ}\ \mathrm{Angle}\ \mathrm{of}\ \mathrm{displacement}\ \left(\alpha \right)=-{\tan}^{-1}\frac{\left(\mathrm{Depth}\ 1-\mathrm{VUJ}\right)-\left(\mathrm{Depth}\ 2-\mathrm{VUJ}\right)}{\left(\mathrm{Height}\ 1-\mathrm{VUJ}\right)-\left(\mathrm{Height}\ 2-\mathrm{VUJ}\right)} $$

Depth 1-VUJ = Preoperative depth of the VUJ (mm); Depth 2-VUJ = Postoperative depth of the VUJ (mm); Height 1-VUJ = Preoperative height of the VUJ (mm); Height 2-VUJ = Postoperative depth of the VUJ (mm).

The postoperative position of the VUJ is always inferior to the preoperative position i.e. the VUJ is deeper within the pelvis following RP surgery. The postoperative position of the VUJ can be either anterior or posterior relative to the preoperative position. When Depth 1-VUJ > Depth 2-VUJ, the angle of displacement of the VUJ following RP occurs in an anterior direction. When Depth 1-VUJ < Depth 2-VUJ, the angle of displacement following RP is in a posterior direction. When D1-VUJ > D2-VUJ the angle of displacement was recorded as a negative angle to describe the angle of displacement in an anterior direction. When Depth 1-VUJ < Depth 2-VUJ, the angle of displacement was recorded as a positive angle to describe the angle of displacement.

### Statistical analysis

Means and standards deviations (SD) and/or medians with interquartile range (IQR) and range were used to describe continuous variables. Categorical variables were summarised using count with percentage (%) for each group. Kolmogorov-Smirnov tests [15] were used to check the normality of the displacement measures and the angle of displacement. Because of the multiple tests a more stringent significance level of 0.01 was used for these tests. Interrater reliability of the VUJ, PMU and ARJ displacement measurements and the VUJ angle of displacement measurement was performed using a two-way mixed-effects agreement intra-class correlation coefficient (ICC) with 95% confidence intervals (CI). Test-retest (intrarater) reliability was calculated using two-way random-effects consistency ICC with 95% CI for all displacement measures of the VUJ, PMU and ARJ for one observer between two different days. Calculated ICC’s were interpreted as: poor < 0.5, moderate 0.5 to 0.75, good 0.75 to 0.9 and excellent > 0.9 [16]. Using methods described by Walter et al. [17], with two raters (or two repeated measurements), a 5% significance level and 80% power to detect a hypothesised poor reliability (0.4, say) versus an alternative good reliability (0.8, say), a minimum sample size of 16 patients was needed. We decided to use a slightly larger sample size of 20 patients. The statistical software IBM SPSS version 24 was used for all analyses.

## Results

Twenty patients, all of whom had laparoscopic RP underwent both preoperative and three-month postoperative MRI scans. The patient characteristics are listed in Table 1.

**Table 1:** Patient Characteristics (*n* = 20)

	Mean ± SD	Median (IQR)	Range
Age (years)	66.4 ± 5.5	66.5 (63.0, 71.5)	53.0–74.0
Preoperative PSA (ng.mL^−1^)	15.4 ± 23.4	7.0 (4.2, 13.4)	3.0–94.0
Prostate volume (mL)	30.7 ± 13.2	27.5 (20.6, 32.4)	16.5–69.3
Preoperative MUL (mm)	13.4 ± 3.5	12.8 (12.2, 15.4)	6.8–20.5
Gleason Grade Group	n (%)		
1	7 (35)		
2	4 (20)		
3	6 (30)		
4	1 (5)		
5	2 (10)		

*SD* Standard deviation, *IQR* Interquartile range, *MUL* Membranous urethral length

The VUJ, PMU and the ARJ displacement measurements (mm) (mean ± SD) and the angle of displacement of the VUJ (degrees) (mean ± SD) results for the PCL, HOR-1 and HOR-2 axis systems are presented in Table 2. None of these measures showed evidence of non-normality using the more stringent significance level because of multiple testing (*p* > 0.02).

**Table 2:** The mean ± SD displacement (mm) of VUJ, PMU and ARJ and VUJ angle of displacement (degrees) measured using the pubococcygeal axis (PCL), HOR-1 and HOR-2 axis reference systems in 20 men following RP

	VUJ Displacement (mm)		PMU Displacement (mm)		ARJ Displacement (mm)		VUJ Angle of displacement (degrees)	
	Day 1	Day 2	Day 1	Day 2	Day 1	Day 2	Day 1	Day 2
PCL axis system								
Observer 1	34.1 ± 4.3	33.7 ± 4.7	4.1 ± 2.1	4.0 ± 1.6	3.5 ± 2.5	4.1 ± 2.6	−16.9 ± 11.6	−16.3 ± 11.4
Observer 2	33.9 ± 4.9		4.7 ± 2.0		4.4 ± 2.5		−16.8 ± 11.0	
HOR-1 axis system								
Observer 1	34.9 ± 4.4	34.4 ± 4.6	4.2 ± 2.3	4.6 ± 2.2	4.0 ± 2.0	3.7 ± 1.9	13.9 ± 10.7	12.7 ± 11.0
Observer 2	34.7 ± 5.0		5.3 ± 2.3		5.1 ± 2.6		11.8 ± 12.2	
HOR-2 axis system								
Observer 1	34.8 ± 4.3	34.6 ± 4.9	4.0 ± 2.0	3.7 ± 1.9	3.9 ± 2.2	4.6 ± 2.5	13.0 ± 11.1	13.6 ± 11.1
Observer 2	34.8 ± 5.1		4.2 ± 2.2		4.4 ± 2.44		13.6 ± 10.8	

*VUJ* Vesico-urethral junction, *PMU* Proximal membranous urethra, *ARJ* anorectal junction, *PCL axis system*: Pubococcygeal line axis system, *HOR-1 axis system*: Horizontal line 1 axis system, *HOR-2 axis system*: Horizontal line 2 axis system

The ICC (95% CI) results for the PCL, HOR-1 and HOR-2 axis systems are presented in Table 3. The PCL axis measurement system demonstrated good to excellent intrarater and interrater reliability for the VUJ and PMU displacement and the VUJ angle of displacement measurements. The intrarater and interrater reliability of the ARJ displacement measures was the most variable with intervals ranging from poor to excellent reliability across the three axis systems. Table 4 shows that the HOR-1 measurements are consistently more variable than the PCL and HOR-2 measurements except for the VUJ displacement. Small differences in variability were observed between PCL and HOR-2 measurements though the variability in PCL measurements was smallest of all of the measurement systems.

**Table 3:** Intra- and interrater reliability with 95% confidence interval (CI) for the measurement of displacement of the VUJ, PMU and ARJ (mm) and the VUJ angle of displacement (degrees)

	VUJ Displacement (mm)	PMU Displacement (mm)	ARJ Displacement (mm)	VUJ Angle of displacement (degrees)
Intrarater Reliability ICC (95%CI) Day 1 vs Day 2				
PCL axis system	0.97 (0.93, 0.99)	0.93 (0.84, 0.97)	0.78 (0.53, 0.91)	0.99 (0.98, 1.00)
HOR-1 axis system	0.96 (0.90, 0.99)	0.80 (0.58, 0.92)	0.71 (0.38, 0.88)	0.96 (0.89, 0.98)
HOR-2 axis system	0.96 (0.91, 0.99)	0.89 (0.74, 0.95)	0.78 (0.51, 0.91)	0.99 (0.97, 1.00)
Interrater Reliability ICC (95%CI) Observer 1 vs Observer 2				
PCL axis system	0.97 (0.93, 0.99)	0.89 (0.75, 0.96)	0.84 (0.63, 0.93)	0.99 (0.96, 0.99)
HOR-1 axis system	0.94 (0.85, 0.98)	0.64 (0.26, 0.83)	0.60 (0.22, 0.82)	0.95 (0.87, 0.98)
HOR-2 axis system	0.94 (0.86, 0.98)	0.85 (0.67, 0.94)	0.76 (0.48, 0.9)	0.98 (0.95, 0.99)

*VUJ* Urethrovesicular junction, *PMU* Proximal membranous urethra, *ARJ* anorectal junction, *ICC* Intra-class correlation coefficient, *CI* confidence interval, *PCL* axis system: Pubococcygeal line axis system, *HOR-1 axis system*: Horizontal line 1 axis system, *HOR-2 axis system*: Horizontal line 2 axis system

**Table 4:** Mean ± SD of the differences between and within raters for the measures of displacement of the VUJ, PMU and ARJ (mm) and the VUJ angle of displacement (degrees)

	VUJ Displacement mm	PMU Displacement (mm)	ARJ Displacement (mm)	VUJ Angle of displacement (degrees)
Mean difference within Observer 1 measurements. Day 1 vs Day 2				
PCL axis system	−0.3 ± 1.0	−0.1 ± 0.7	0.6 ± 1.6	0.6 ± 1.5
HOR-1 axis system	− 0.5 ± 1.1	0.4 ± 1.4	0.9 ± 1.7	−1.2 ± 3.1
HOR-2 axis system	−0.2 ± 1.3	−0.3 ± 0.9	0.6 ± 1.5	0.8 ± 1.3
Mean difference between Observer 1 and Observer 2 measurements				
PCL axis system	0.2 ± 1.1	0.5 ± 0.9	0.8 ± 1.4	0.0 ± 2.0
HOR-1 axis system	0.2 ± 1.6	1.1 ± 2.0	0.8 ± 2.3	−2.0 ± 3.7
HOR-2 axis system	0.0 ± 1.6	−0.2 ± 1.3	0.5 ± 1.6	0.8 ± 2.2

*VUJ* Vesiculo-urethral junction, *PMU* Proximal membranous urethra, *ARJ* anorectal junction, *PCL* axis system: Pubococcygeal line axis system, *HOR-1 axis system* Horizontal line 1 axis system, *HOR-2 axis system* Horizontal line 2 axis system

## Discussion

This study has addressed the reliability of three new methods that can potentially be used to calculate the postoperative displacement of the VUJ, PMU and ARJ and the VUJ angle of displacement via the comparative measurement of pre- and postoperative MRI scans. Excellent reliability was demonstrated for the VUJ and PMU displacement measures and the VUJ angle of displacement for the PCL and HOR-2 measurement systems. The reliability of ARJ displacement measurements was highly variable regardless of the measurement system that was selected.

The displacement of the VUJ and PMU is an outcome of RP surgery. The newly formed urethrovesical anastomosis is created when traction is applied to the VUJ and PMU. The measurement of the VUJ and PMU anatomical change in position within the pelvis was possible by the incorporation of an X, Y axis system using standardized and previously reported pelvimetry reference lines [13, 14]. To achieve reliable and reproducible comparative measurement of the lower urinary tract system [18] we used fixed anatomical structures that serve as stable reference points. The pelvic bony structures, including the pubic symphysis and the sacrococcygeal joint appear as clearly defined anatomical structures on T2W midsagittal MRI images.

The PCL, HOR-1 and HOR-2 MRI measuring systems all use the inferior edge of the pubic symphysis as a common stable bony point of reference [13, 14]. The PCL MRI measuring system has an advantage compared to the HOR-1 and HOR-2 MRI measuring systems by having the sacrococcygeal joint as the second stable bony point of reference. This may have been a contributing factor to the more consistent and reliable PCL measurements. The PCL has been previously used to measure the position of pelvic organs in men prior to and following RP and is related to urinary incontinence outcomes [12]. A potential limitation to the application of the PCL MRI measuring system occurs when the field of view for pre- and postoperative pelvic MRI images does not capture a clearly defined sacrococcygeal joint. Anatomical variations in the structure of the sacrococcygeal region, including fusion of the sacrococcygeal joint may potentially make accurate identification problematic [19]. The HOR-2 measurement system could be then used to provide an alternate approach when use of the PCL measurement system is not possible.

We acknowledge some limitations of this investigation. This study was a retrospective design involving a sample of twenty patients from a single facility in Korea and single surgeon series. While the sample size was small, a larger than required sample size was used. Increasing the sample size further was not possible due to time constraints and limited resources. The patient characteristics from this sample were similar to those observed in other studies [20–22] suggesting a reasonable range of anatomical variability was observed however it is possible that the results are not generalisable beyond the population studied. Larger studies that include, in particular, more patients with large prostate volumes, may provide valuable additional information regarding all measuring systems.

## Conclusions

Comparative measurement of pre- and postoperative MRI scans can be used to assess the reliability of measurement of the displacement of the vesico-urethral junction and proximal membranous urethra, and the angle of displacement of the vesico-urethral junction in men following radical prostatectomy. In this study of 20 Korean patients with median prostate volume 27.5 mL and maximum volume 70 mL, the pubococcygeal line measurement system was preferred, providing the smallest differences in variability to quantify of the postoperative displacement of the anatomical structures that can affect urinary incontinence and erectile dysfunction following RP. Further studies should validate these results in patients with large prostate volumes.

## Abbreviations

ARJ: Anorectal junction
BU: Penile bulb
CI: Confidence interval
Depth 1: Preoperative depth from either the PCL, HOR-1 or HOR-2 reference line
Depth 2: Postoperative depth from either the PCL, HOR-1 or HOR-2 reference line
Height 1 – ARJ: Preoperative height of the anorectal junction from either the PCL, HOR-1 or HOR-2 reference lines
Height 1 – PMU: Preoperative height of the proximal membranous urethra from either the PCL, HOR-1 or HOR-2 reference lines
Height 1 – VUJ: Preoperative height of the VUJ from either the PCL, HOR-1 or HOR-2 reference line
Height 1: Preoperative perpendicular distance from the PCL, HOR-1 or HOR-2 reference lines
Height 2 – ARJ: Postoperative height of the anorectal junction from either the PCL, HOR-1 or HOR-2 reference lines
Height 2 – PMU: Postoperative height of the proximal membranous urethra from either the PCL, HOR-1 or HOR-2 reference lines
Height 2 – VUJ: Postoperative height of the VUJ from either the PCL, HOR-1 or HOR-2 reference line
Height 2: Postoperative perpendicular height from either the PCL, HOR-1 or HOR-2 reference lines
HOR-1: Midsagittal horizontal line axis 1
HOR-2: Midsagittal horizontal line axis 2
ICC: Intraclass correlation coefficient
MRI: Magnetic resonance imaging
MUL: Membranous urethral length
PCL: Pubococcygeal line
PMU: Proximal membranous urethra
PV: Prostate volume
VUJ: Vesico-urethrol junction
